# Polymyxin B_1_ and E_2_ From *Paenibacillus polymyxa* Y-1 for Controlling Rice Bacterial Disease

**DOI:** 10.3389/fcimb.2022.866357

**Published:** 2022-03-28

**Authors:** Wenshi Yi, Chao Chen, Xiuhai Gan

**Affiliations:** ^1^ State Key Laboratory Breeding Base of Green Pesticide and Agricultural Bioengineering, Key Laboratory of Green Pesticide and Agricultural Bioengineering Ministry of Education, Guizhou University, Guiyang, China; ^2^ School of Chemistry and Materials Science, Guizhou Education University, Guiyang, China

**Keywords:** *Paenibacillus polymyxa* Y-1, polymyxin, rice bacterial blight, antibacterial activity, phenylpropanoid biosynthesis

## Abstract

To discover novel microbial pesticide for controlling rice bacterial disease, polymyxin B_1_ and E_2_ were firstly isolated from the supernatant of fermentation broth of *Paenibacillus polymyxa Y-1* by bioactivity tracking separation. It is shown that polymyxin B_1_ and E_2_ had remarkable *in vitro* inhibitory activities to *Xanthomonas oryzae* pv. *oryzae* (*Xoo*) and *Xanthomonas oryzae* pv. *oryzicola* (*Xoc*) with the EC_50_ values of 0.19 μg/ml and 0.21 μg/ml against *Xoo*, and 0.32 μg/ml and 0.41 μg/ml against *Xoc*, respectively, which were better than those of Zhongshengmycin (0.31 μg/ml and 0.73 μg/ml) and Bismerthiazol (77.48 μg/ml and 85.30 μg/ml). Polymyxins B_1_ and E_2_ had good protection and curative activities against rice bacterial leaf blight (BLB) and rice bacterial leaf streak (BLS) *in vivo*. The protection and curative activities of polymyxins B_1_ (45.8 and 35.8%, respectively) and E_2_ (41.2 and 37.0%, respectively) to BLB were superior to those of Zhongshengmycin (34.8 and 29.8%, respectively) and Bismerthiazol (38.0 and 33.5%, respectively). Meanwhile, the protection and curative activities of polymyxins B_1_ (44.8 and 39.8%, respectively) and E_2_ (42.9 and 39.9%, respectively) to BLS were also superior to those of Zhongshengmycin (39.7 and 32.0%, respectively) and Bismerthiazol (41.5 and 34.3%, respectively). Polymyxin B_1_ exerted the anti-pesticide properties *via* destroying the cell integrity of *Xoo*, reducing its infectivity and enhancing rice resistance against pathogens through activating the phenylpropanoid biosynthesis pathway of rice. It is indicated that polymyxin B_1_ and E_2_ were potential microbial pesticides for controlling rice bacterial disease.

## Introduction

Plant bacterial diseases are caused by a large number of microorganisms, leading to a substantial economic loss of crops worldwide each year. Two important rice bacterial diseases, rice bacterial leaf streak (BLS) and rice bacterial blight (BLB) caused by *Xanthomonas oryzae* pv. *oryzicola* (*Xoc*) and *Xanthomonas oryzae* pv. *oryzae* (*Xoo*), seriously affect the yield and quality of rice. BLB and BLS result in at least 80 and 40% yield loss annually, respectively (Huang et al., 1997; Li et al., 2012; Jiang et al., 2020). The long-term use of traditional bactericides like Bismerthiazol, Zinc thiazole, and Thiodiazole copper causes drug resistance and adverse events on the safety of the environment (Wang et al., 2019). However, microbial pesticide with low toxicity and easy metabolism properties has been a hot research topic in recent years. As a successful microbial pesticide, Zhongshengmycin has been widely used to control plant bacterial diseases, although its control efficacy is relatively low (Wang et al., 2021). Therefore, it is urgent to discover the alternative to conventional antibiotics with high efficacy, environmental friendliness and low toxicity.

*Dendrobium nobile* (*D. nobile*) is a medicinal and edible plant native to China. It belongs to the *Orchidaceae*, and is rich in endophytic bacteria. *Paenibacillus polymyxa* (*P. polymyxa*) is one of important endophytic bacteria of *D. nobile* with various physiological activators. Among them, peptide antibiotics, a kind of main secondary metabolites derived from various species of *P. polymyxa*, present an acceptable inhibitory effect against pathogens, namely, polymyxin (Landman et al., 2008; Deng et al., 2011; Niu et al., 2013), saltavidin and gavaserin (Pichard et al., 1995), gatavalin (Nakajima et al., 1971), jolipeptin (Ito and Koyama, 1972), and fusaricidin (Kajimura and Kaneda, 1996; Kajimura and Kaneda, 1997). Five polymyxins (polymyxin A to E) have been widely analyzed and applied as antimicrobial agents. Meanwhile, there are some other metabolites produced from *P. polymyxa* like cytokinins, auxins, chitinase, and hydrolase, which can promote plant growth, improve plant utilization of nutrients, and induce plant systemic resistance (Mavingui and Heulin, 1994; Lebuhn et al., 1997; Pham et al., 1998; Timmusk et al., 1999; Budi et al., 2000). Recently, *P. polymyxa* was used to control plant pathogens, namely, *Xanthomonas campestris* and *X. axonopodis* (Hahm et al., 2012; Mageshwaran et al., 2012). However, anti-plant pathogens metabolites of *P. polymyxa* have been rarely reported.

Our previous study, an endophytic bacterium of *D. nobile* living in the Chishui County of Guizhou Province in China, *P. polymyxa* Y-1 was firstly isolated. Meanwhile, three pairs of fusaricidin-type compounds were isolated from *P. polymyxa* Y-1 and their antifungal activities against *Pestalotiopsis* were assessed, and showed the inhibitory effects on energy generation and amino acid biosynthesis of *Pestalotiopsis* (Yang et al., 2018). In the current study, to further discover the anti-plant bacterial metabolites from *P. polymyxa*, two polymyxin metabolites of *P. polymyxa* were isolated by bioactivity tracking separation and their anti-plant bacterial activities were evaluated. The results showed excellent anti-plant bacterial activities of polymyxin B_1_ and E_2_. Furthermore, morphological, conductivity, biofilm, exopolysaccharides (EPS) and differentially expressed genes in polymyxin B_1_ were explored to clarify the potential mechanisms.

## Materials and Methods

### Strains and Culture Conditions

*P. polymyxa* Y-1 was isolated and identified in our previous study (Yang et al., 2018) and cultured at 28°C in potato dextrose broth (PDB) medium and stored at –80°C in Luria-Bertani with 30% (v/v) glycerol. The pathogenic bacterial strain was cultured in 500 ml of PDB at 28°C until bacteria PDB medium has grown into the logarithmic phase. The supernatant was inoculated into 30 L of PDB medium and grown for 14 days until the OD_595_ reached more than 1.1. The fermentation broth was centrifuged at 8,000 rpm for 30 min at room temperature, and then collected the supernatant for testing the antibacterial activity and isolating the antibacterial metabolites.

### Antibacterial Activity Assay

The inhibitory effects of fermentation broth and metabolites of *P. polymyxa* Y-1 against *Xoo* and *Xoc* were examined by paper disk method and turbidimetric method, respectively (Dalgaard et al., 1994; Happy, 2017). Then, the protective and curative activities of metabolites to BLS and BLB were measured in potted plants by Schaad’s method (Li et al., 2018). Bismerthiazol (20% wettable powder) and Zhongshengmycin (12% wettable powder) were served as positive controls. The EC_50_ values and the *in vivo* curative and protective effect of metabolites were analyzed using SPSS 17.0.

### Metabolite Isolation and Structure Identification

**Figure 1:**
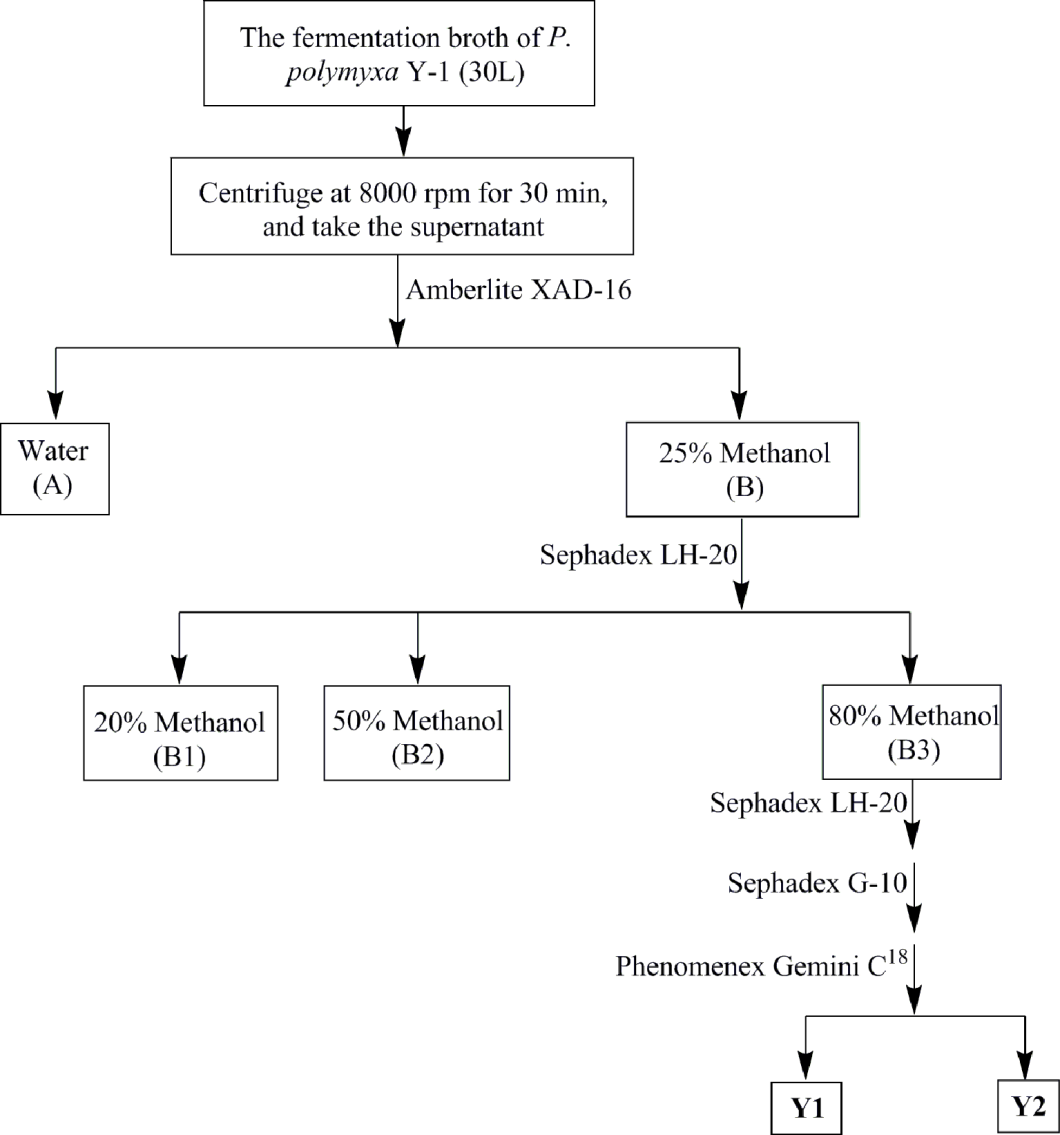
Purification flow chart of the antibacterial compounds of *Paenibacillus polymyxa* Y-1.

The antibacterial metabolites were isolated from *P. polymyxa* Y-1 supernatant by bioactivity tracking separation (**Figure 1**). The supernatant was separated by an Amberlite XAD-16 column eluted with CH_3_OH−H_2_O (0:100, 25:75, 50:50, 80:20, and 100:0 in turn, v/v, 400 ml each) and four fractions (Fr.A to Fr.D) were obtained. The Fr.B (4.3 g) was purified with Sephadex LH-20 column eluted with 20, 50, and 80% CH_3_OH–H_2_O in turn (50 ml each), and the same segments were enriched and concentrated to afford Fr.B1 to Fr.B3. The Fr.B3 (816.2 mg) was purified with Sephadex LH-20 and Sephadex G-10 column eluted with methanol and H_2_O for separating the active fractions, respectively. Finally, the active fraction was further purified by HPLC eluted with 30% CH_3_CN in 0.1% trifluoroacetic acid H_2_O, and Y1 (65.4 mg) and Y2 (78.2 mg) were obtained. The structures were identified by ^1^H NMR, ^13^C NMR, and HRMS.

### Scanning Electron Microscopy (SEM) Detection

The *Xoo* was inoculated into 20 ml of nutrient broth (NB) medium, and cultured to the logarithmic phase (Nollin and Borgers, 1975). The fermentation liquid was centrifuged and *Xoo* cells in the precipitant were washed, re-suspended in PBS (pH = 7.2) and treated with the active metabolite at 10, 5, and 1 µg/ml for 10 h at 28°C, respectively, with Tween buffer as the negative control. Ten hours later, *Xoo* cells were washed in PBS and fixed by the 2.5% glutaraldehyde overnight at 4°C. Subsequently, *Xoo* cells were sequentially dehydrated in 10, 30, 50, 70, 90, and 100% ethanol. Samples were finally freeze-dried, coated, and visualized using Nova Nano SEM 450.

### Conductivity Detection

The *Xoo* was inoculated into 20 ml of NB medium, and cultured to the logarithmic phase. The fermentation liquid was centrifuged and *Xoo* cells in the precipitant were washed three times with sterile water, and re-suspended in 20 ml of sterile water. Subsequently, *Xoo* cells were treated with the active metabolite at 10, 5, and 1 µg/ml, respectively, with Tween 20 solution as the negative control. The conductivity at 0, 30, 60, 120, 180, 240, 300, 360, and 420 min were measured, respectively. Finally, the suspension was boiled for 10 min to kill *Xoo*, followed by detection of the conductivity. The experiment was repeated in triplicate.

### Biofilm Formation Detection

Approximately 200 µl of *Xoo* suspension in the logarithmic phase was added to 2 ml of NB medium. The mixture in NB medium was prepared at the active metabolite concentration of 10, 5, and 1 µg/ml, respectively, which was cultured at 28°C for 5 d. The effects of biofilm formation treated with active metabolite were performed according to the previous method (Pan et al., 2018). Tween 20 solution was used as the negative control. The experiment was repeated in triplicate.

### EPS Detection

Approximately 150 µl of *Xoo* suspension in the logarithmic phase was added to 15 ml of NB medium to prepare the mixture containing 10, 5, and 1 µg/ml active metabolites, and cultured for 72 h at 28°C with 180 rpm shaking. The content of EPS was detected by previously reported method (Denny, 1995). Tween 20 solution was used as the negative control. The experiment was repeated in triplicate.

### Analysis of Differentially Expressed Proteins (DEPs)

Under greenhouse conditions, Fengyouxiangzhan (seeds were purchased from the Guizhou Seed Management Station) rice was cultivated for 8 weeks, and 200 μg/ml active metabolite solutions were uniformly sprayed onto rice leaves of the treatment group until dripping down, whereas Tween 20 solution was used as control groups. One day after spraying, *Xoo* in the logarithmic phase was inoculated onto rice leaves with scissors method. Then, the rice plants were cultivated in greenhouse at 28°C and 90% RH. Five days after inoculation, rice leaves were extracted and enzymatically digested for LC–MS/MS (Bin et al., 2020). DEPs were analyzed using the gene ontology (GO) annotation (http://www.geneontology.org/), and the Kyoto Encyclopedia of Genes and Genomes (KEGG) annotation (http://www.genome.jp/Pathway). Then, the databases were searched using the Uniprot software (http://www.uniprot.org/) (Gene, 2004).

### Determination of Defensive Enzyme Activities

The activities of dehydrogenase (CAD) and peroxidase (POD) in rice leaves were determined on the 1st, 3rd, and 5th days after inoculation by the instructions of the manufacturer using commercial enzyme assay reagent kits (Beijing Solarbao Life Sciences, China).

### Parallel Reaction Monitoring (PRM)

Rice leaves were extracted and enzymatically digested, and then analyzed by LC–MS/MS. Label-free proteomics samples were taken from the same batch of rice leaves. MS data were processed using Skyline (v.3.6). Peptide parameters were set as follows: Trypsin [KR/P]; Max missed cleavage = 0; Peptide length = 7–25 amino acid residues; Fixed modification of cysteine alkylation. Transition parameters were set as follows: Precursor charges = 2, 3; Ion charges = 1, 2; Ion types = b, y, p; Product ions = ion 3 to last ion I; Ion match tolerance = 0.02 Da (Tsuchiya et al., 2013; Yu et al., 2014; Du et al., 2015).

## Results and Discussion

### Purification and Structure Identification of Metabolites

**Figure 2:**
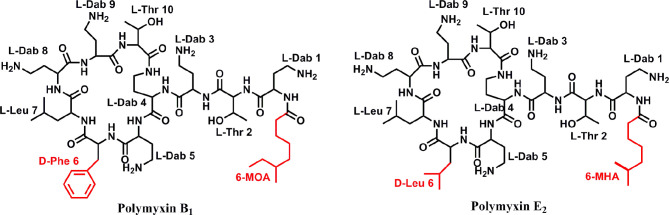
Chemical structure of antibacterial compounds polymyxin B_1_ and E_2_.

A series of bioassay results showed that the supernatant of fermentation broth from *P. polymyxa* Y-1 presented remarkable antibacterial activities against *Xoo* (33.3%) and *Xoc* (31.5%) (**Figure S1**). After purification, two antibacterial metabolites coded Y1 and Y2 were isolated for the supernatant. The ^1^H NMR (600 MHz, D_2_O) chemical shifts of antibacterial active metabolites Y1, chemical shifts of *δ* 7.36–7.23 (m, 5H) indicated the existence of aromatic groups. Meanwhile, the chemical shift of *δ* 4.58–4.39 (m, 5H), 4.34–4.14 (m, 7H) indicated the presence of H^α^ and Thr-H^β^. The ^13^C NMR chemical shifts of *δ* 177.7, 175.1, 173.6, 173.4, 173.1, 173.0, 172.9, 172.4, 171.9, 171.8, and 171.5 indicated the presence of carbonyl carbon, and the chemical shift of *δ* 135.3, 129.0, 129.0, 128.9, 128.9, and 127.4 confirmed the presence of aromatic groups. HRMS exhibited ion peaks [M + H]^+^ at m/z 1,203.69739, by ^1^H NMR, ^13^C NMR, and HRMS analysis, the compounds were identified as polymyin B_1_ (calculated for C_56_H_99_O_13_N_16_, [M + H]^+^, 1,203.75720). Similarly, the ^1^H NMR (600 MHz, D_2_O) chemical shifts of antibacterial active metabolites Y2, *δ* 4.55–4.50 (m, 3H), 4.41–4.35 (m, 3H), 4.30–4.22 (m, 5H), and 4.18 (d, *J* = 4.78 Hz, 1H) suggested H^α^ and Thr-H^β^. The ^13^C NMR chemical shifts of *δ* 177.7, 175.0, 174.9, 173.5, 173.1, 172.9, 172.9, 172.4, 172.2, 171.9, and 171.5 represented the carbonyl carbon. HRMS exhibited ion peaks [M + H]^+^ at m/z 1,155.76685, by ^1^H NMR, ^13^C NMR, and HRMS analysis, and the compounds were identified as polymyin E_2_ (calculated for C_52_H_99_O_13_N_16_, [M + H]^+^, 1,155.75720) (**Figure 2**) (Ikai et al., 1998; Bruch et al., 1999; Pristovsek and Kidric, 1999; Orwa et al., 2001; Cui et al., 2018) (**Supplementary Data**). The study for the first time reported the structures of polymyins from *P. polymyxa* Y-1.

### *In Vitro* Antibacterial Activity Assay

The *in vitro* antibacterial activities of polymyxin B_1_ and E_2_ against *Xoo* and *Xoc* were evaluated by the turbidimeter method, and the results are listed in **Table 1**. It is shown that polymyxin B_1_ and E_2_ exhibited excellent antibacterial activities (100%) against *Xoo* and *Xoc* at the concentration of 200 and 100 μg/ml, respectively, which were similar to Zhongshengmycin, but superior to Bismerthiazol (74.1 and 53.1%, 81.0 and 65.24%, respectively). The antibacterial activities of polymyxin B1 and E2 were confirmed with the EC_50_ values against *Xoo* of 0.19 and 0.21 μg/ml, respectively, and 0.32 and 0.41 μg/ml against *Xoc*, respectively, which were better than those of Zhongshengmycin (0.31 and 0.73 μg/ml) and Bismerthiazol (77.48 and 85.30 μg/mL) against *Xoo* and *Xoc*, respectively. It is indicated that polymyxin B_1_ and E_2_ can be considered as new antibacterial agents.

**Table 1:** Antibacterial Activity of Polymyxin B_1_ and E_2_ against *Xoo* and *Xoc*.

Metabolite	*Xoo*			*Xoc*		
	200 μg/ml (%)	100 μg/ml (%)	EC_50_ (μg/ml)	200 μg/ml (%)	100 μg/ml (%)	EC_50_ (μg/ml)
Polymyxin B_1_	100	100	0.19 ± 0.09	100	100	0.32 ± 0.05
Polymyxin E_2_	100	100	0.21 ± 0.04	100	100	0.41 ± 0.04
Zhongshengmycin	100	100	0.31 ± 0.06	100	100	0.73 ± 0.07
Bismerthiazol	74.1 ± 1.6	53.1 ± 2.7	77.48 ± 2.62	81.0 ± 2.5	65.2 ± 3.7	85.30 ± 1.06

### *In Vivo* Antibacterial Activity Assay

**Figure 3:**
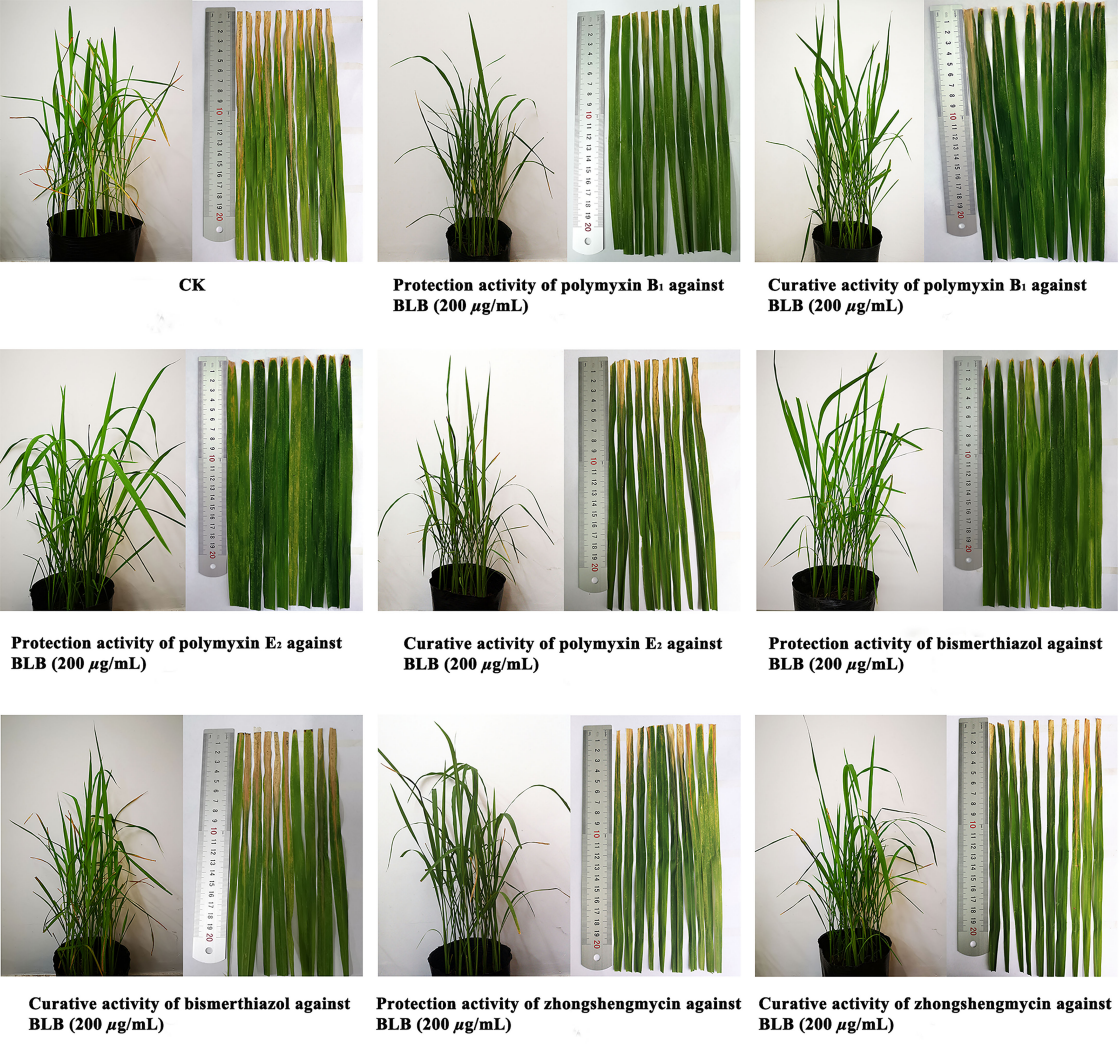
Protection activity and curative activity of polymyxin B_1_ and E_2_ against BLB under greenhouse conditions at 200 μg/ml.

**Figure 4:**
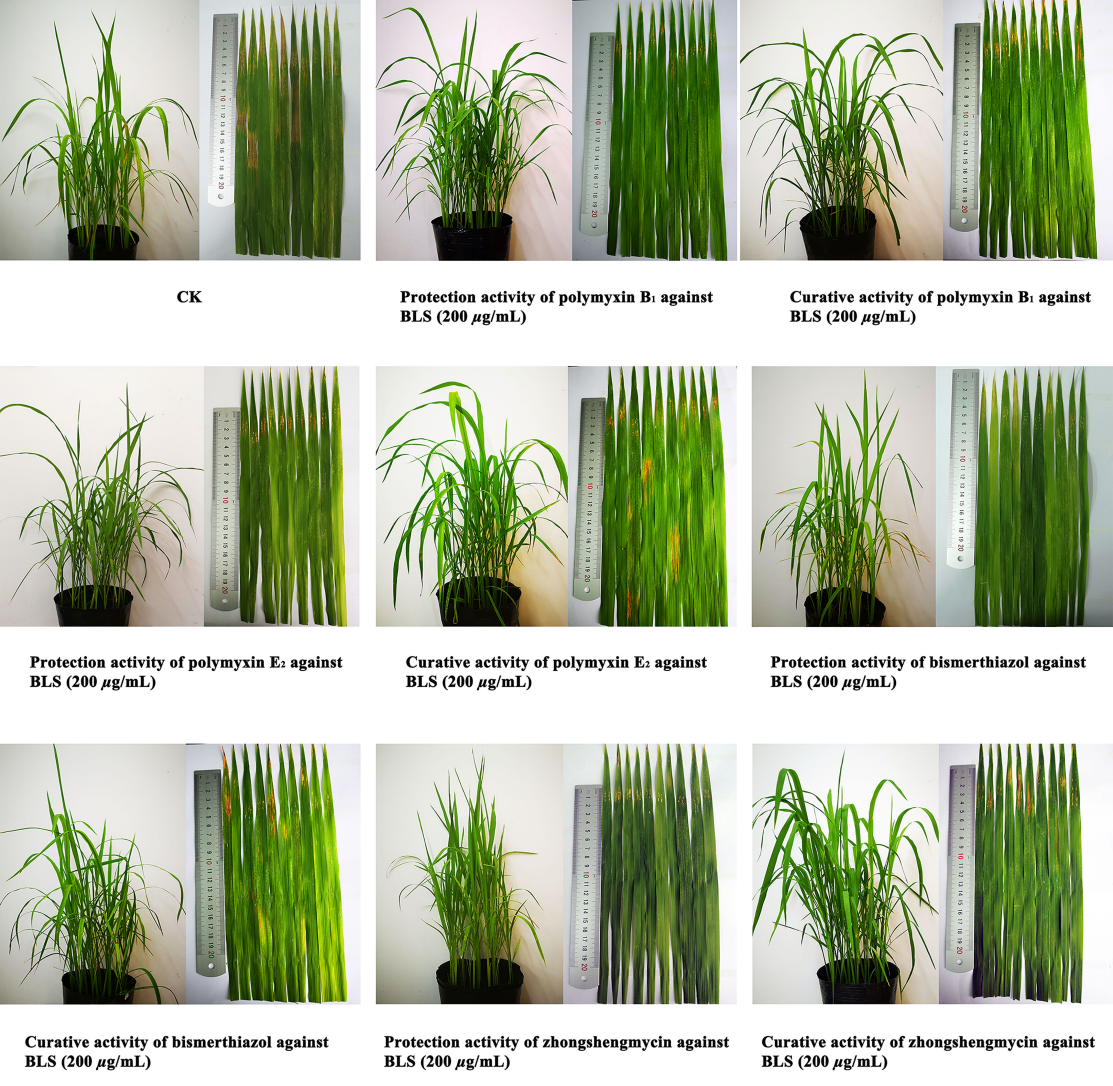
Protection activity and curative activity of polymyxin B_1_ and E_2_ against BLS under greenhouse conditions at 200 μg/ml.

As shown in **Table 2** and **Figure 3**, polymyxin B_1_ (45.8%) and E_2_ (41.2%) exhibited a better protection activity against BLB than that of Zhongshengmycin (34.8%) and Bismerthiazol (38.0%) under the greenhouse condition at 200 μg/ml. Compared with the curative activity of Zhongshengmycin (29.8%) and Bismerthiazol (33.5%), polymyxin B_1_ (35.8%) and E_2_ (37.0%) also showed an excellent curative effect to BLB. In addition, polymyxin B_1_ (44.8 and 39.8%) and E_2_ (42.9 and 39.9%) also exhibited excellent protection and curative activities against BLS under the greenhouse condition at 200 μg/ml, which were better than those of Zhongshengmycin (39.7 and 32.0%) and Bismerthiazol (41.50 and 34.3%) (**Table 3** and **Figure 4**). The results indicated that polymyxin B_1_ and E_2_ significantly reduced the occurrence of BLB and BLS diseases.

**Table 2:** Effect of Polymyxin B_1_ and E_2_ against BLB under Greenhouse Conditions at 200 μg/ml.

Treatment	Protection activity		Curative activity	
	Infection index (%)	Control efficiency (%)	Infection index (%)	Control efficiency (%)
Polymyxin B_1_	39.1 ± 0.7	45.8 ± 1.4	46.3 ± 2.5	35.8 ± 2.3
Polymyxin E_2_	42.4 ± 1.5	41.2 ± 3.0	49.0 ± 0.6	37.0 ± 0.5
Zhongshengmycin	47.0 ± 0.3	34.8 ± 1.4	50.6 ± 2.3	29.8 ± 1.8
Bismerthiazol	44.6 ± 1.6	38.0 ± 3.2	47.9 ± 0.3	33.5 ± 1.5
CK	72.2 ± 1.3	/	72.2 ± 1.3	/

CK is the blank rice group, its control efficiency is represented by the symbol "/".

**Table 3:** Effect of Polymyxin B_1_ and E_2_ against BLS under Greenhouse Conditions at 200 μg/ml.

Treatment	Protection activity		Curative activity	
	Infection index (%)	Control efficiency (%)	Infection index (%)	Control efficiency (%)
Polymyxin B_1_	40.7 ± 1.6	44.8 ± 1.2	44.5 ± 1.2	39.8 ± 0.6
Polymyxin E_2_	42.1 ± 2.8	42.9 ± 4.3	44.3 ± 1.2	39.9 ± 1.8
Zhongshengmycin	44.6 ± 1.2	39.7 ± 3.3	50.2 ± 0.5	32.0 ± 1.6
Bismerthiazol	43.2 ± 2.5	41.5 ± 4.2	48.4 ± 1.9	34.3 ± 3.9
CK	73.9 ± 2.2	/	73.9 ± 2.2	/

CK is the blank rice group, its control efficiency is represented by the symbol "/".

### SEM Detection

**Figure 5:**
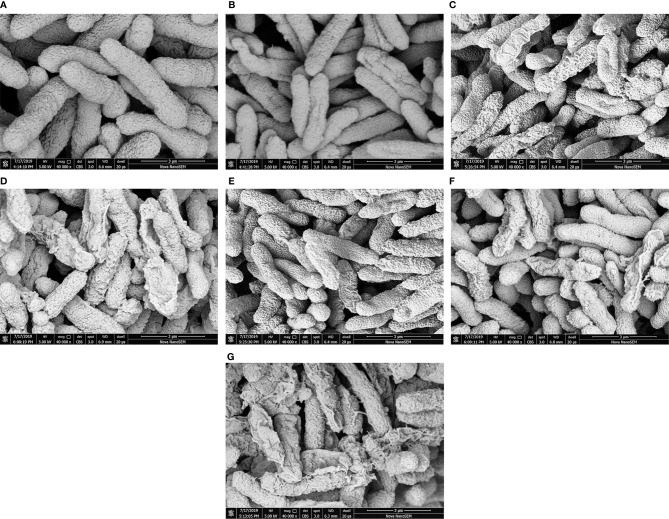
Cell surface morphologies of *Xoo* treated with polymyxin B_1_ and E_2_. **(A)** CK, **(B)** *Xoo* cells treated with polymyxin B_1_ (1 μg/mL), **(C)** *Xoo* cells treated with polymyxin B_1_ (5 μg/mL), **(D)** *Xoo* cells treated with polymyxin B_1_ (10 μg/mL), **(E)** *Xoo* cells treated with polymyxin E_2_ (1 μg/mL), **(F)** *Xoo* cells treated with polymyxin E_2_ (5 μg/mL), **(G)** *Xoo* cells treated with polymyxin E_2_ (10 μg/mL).

The effects of polymyxin B_1_ and E_2_ on *Xoo* cell membranes were examined by SEM. As shown in **Figure 5**, the thallus of the CK groups was plump and uniform on the surface (**Figure 5A**). However, the morphologies of *Xoo* have been deformed and wrinkled (**Figures 5B, E**) after treatment with polymyxin B_1_ and E_2_ at 1 μg/ml, respectively. Meanwhile, the morphologies of *Xoo* were transformed corrugated structure, and partially broken state at 5 μg/ml (**Figures 5C, F**). A pore-like shape on the bacterial surface and a lot of fragmentized bacteria were observed at the concentration of polymyxin B_1_ and E_2_ of 10 μg/ml (**Figures 5D, G**). SEM results confirmed that polymyxin B_1_ and E_2_ could act on cell membranes and damage the cell integrity of *Xoo*.

### Conductivity Detection

**Figure 6:**
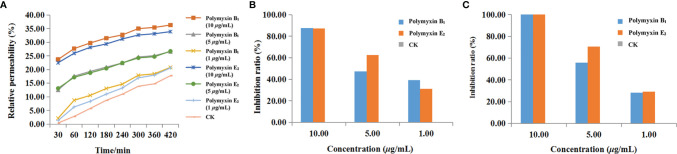
The effects of polymyxin B_1_ and E_2_ on relative conductivity **(A)**, biofilm formation **(B)**, and EPS **(C)** to *Xoo*.

Conductivity was determined to reveal the membrane permeability. As shown in **Figure 6A**, increased effects of polymyxin B_1_ and E_2_ on the cell membrane permeability of *Xoo* with time and concentration were observed. There were significant differences in the treatment concentrations. The relative permeability of polymyxin B_1_ and E_2_ were 23.7 and 22.4% at 10 μg/ml for 30 min, which were 12.4 and 13.1% at 5 μg/ml, and 2.1 and 1.3% at 1 μg/ml, respectively. All of them were significantly higher than those of CK groups (0.4%). In addition, the relative permeability of the bacterial liquid increased with time. Notably, the conductivity rapidly increased within 60 min, which slowly increased with the treatment of polymyxin B_1_ and E_2_. Among them, the relative permeability of the bacterial solution treated with polymyxin B_1_ and E_2_ were much higher than that of the CK group. Seriously damaged cell membrane might be the main reason for the antibacterial effect of polymyxin B_1_ and E_2_.

### Biofilm Formation Detection

The effect of the agent on cell biofilm was determined by crystal violet staining. As shown in **Figure 6B**, the biofilm formation was inhibited after treatment with polymyxin B_1_ and E_2_. The inhibition ratios of polymyxin B_1_ and E_2_ at 10, 5, and 1 μg/ml were 87.56, 47.28, 39.26% and 87.37, 62.78, 31.18%, respectively, which were superior to CK groups. It can be seen that polymyxin B_1_ and E_2_ might act on *Xoo* biofilm and the effect was positively correlated with the concentration.

### EPS Detection

The effects of polymyxin B_1_ and E_2_ on EPS were determined and the results were shown in **Figure 6C**. Polymyxin B_1_ and E_2_ at 10 μg/ml significantly inhibited the production of EPS. Meanwhile, the inhibition ratios of polymyxin B_1_ and E_2_ at 5 μg/ml were 55.93 and 70.42%, respectively, which were 28.19 and 29.34% at 1 μg/ml. It is shown that polymyxin B_1_ and E_2_ had significant inhibitory effects on the production of EPS from *Xoo* and then decreased the pathogenicity of *Xoo*.

### DEPs Analysis

**Figure 7:**
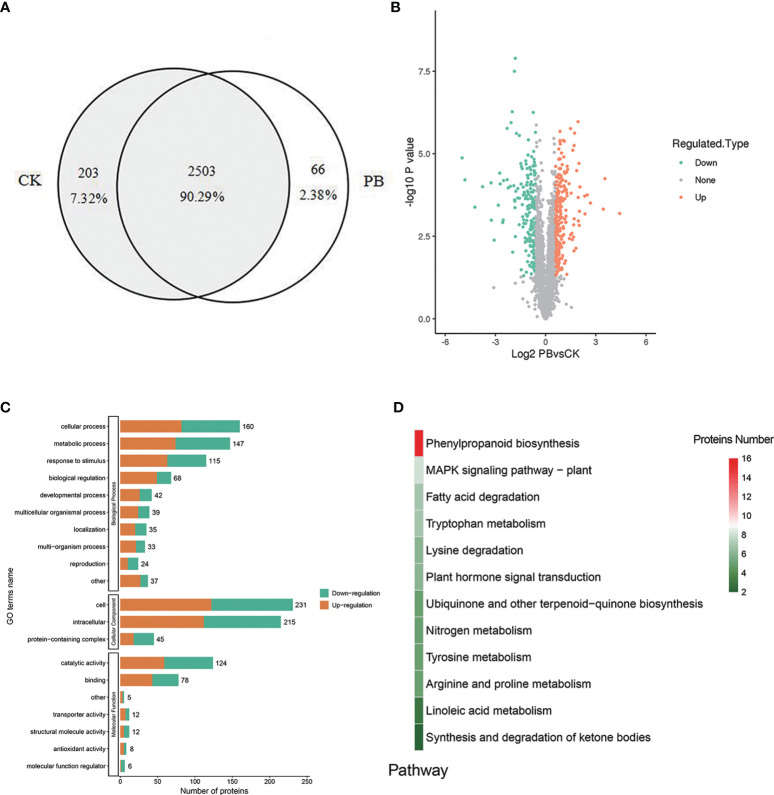
DEPs. **(A)** Venn diagram for proteins identified in the CK + *Xoo* (CK) and PB_1_ + *Xoo* (PB) groups, **(B)** Differential protein volcano map (|log_2_ foldchange| >1, *P <*0.05), **(C)** GO annotation and enrichment analysis of the DEPs between PB_1_ + *Xoo* (PB) and CK + *Xoo* (CK) groups, **(D)** KEGG analysis of the DEPs between PB_1_ + *Xoo* (PB) and CK + *Xoo* (CK) groups.

A total of 2,772 proteins were identified in both polymyxin B_1_ + *Xoo* (PB) groups and CK + *Xoo* (CK) groups, which are shown in the **Supplementary LC–MS data**. There were 2,706 and 2,569 proteins identified in PB and CK groups, respectively, including 203 (7.32%) and 66 (2.38%) specifically expressed proteins, respectively (**Figure 7A**). A total of 460 proteins were DEPs, including 251 (54.57%) upregulated proteins and 209 (45.43%) downregulated ones (**Figure 7B**). GO analysis results showed that biological processes were mainly enriched in “cellular process”, “metabolic process”, “response to stimulus”, and “biological regulation”. Cellular component only enriched in “cell”, “intracellular”, and “protein-containing complex” (**Figure 7C**). Molecular functions were mostly enriched in “catalytic activity” and “binding”. It is demonstrated that polymyxin B_1_ could affect rice proteome in many aspects of plant physiology, namely, induced resistance, metabolic process, and cellular process.

**Figure 8:**
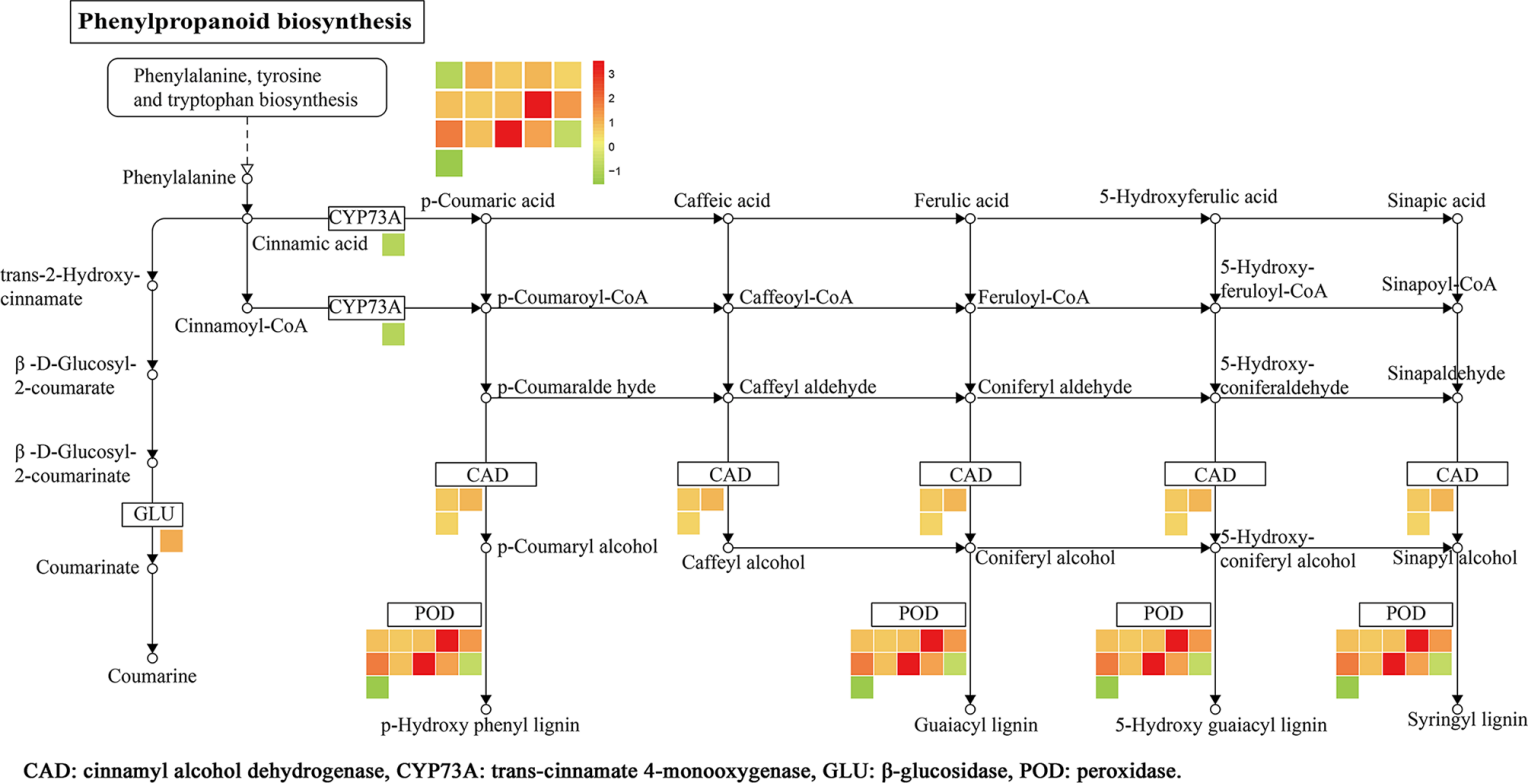
DEPs related to phenylpropanoid biosynthesis in rice leaf. The log_2_ fold change of genes is indicated as a gradient between red (upregulated) and green (downregulated). (1≤ log_2_ fold change ≤−1, *P <* 0.05).

KEGG analysis results showed that a total of 12 pathways were enriched (**Figure 7D**), namely, the phenylpropanoid biosynthesis, MAPK signaling pathway, fatty acid degradation, tryptophan metabolism, lysine degradation, and others. Among them, 16 DEPs was enriched in the phenylpropane biosynthetic pathway, namely, trans-cinnamate-4-monooxygenase (CYP73A), *β*-glucosidase (GLU), CAD, and POD. There were 9 upregulated proteins (A0A0N7KI36, A0A0P0XR31, Q5JMS4, Q94DM2, A2YPX2, Q6ZCC2, Q6K4J4, Q7XSU7, and Q7XSU8) and 2 downregulated ones (A0A0P0V2C2, Q9AS12) involved in the regulation of POD. Meanwhile, CAD (Q8H859, Q6ERW9, and Q6ERW7) and GLU (Q60DX8) proteins were upregulated, but CYP73A (A2Y375) was downregulated (**Figure 8**). It is well-known that POD and CAD are able to regulate the formation of lignin (Hrubcová et al., 2000; Fan et al., 2009; Sui et al., 2019), which can provide structural support, prevent pathogen invasion through a physical barrier and act as defensive components in plants (Weng and Chapple, 2010; Aehle et al., 2011; Labeeuw et al., 2015). Upregulation of CAD and POD can increase the resistance of plants to invaded pathogens and reduce the infection incidence (Chen et al., 2019; Yadav et al., 2020). The results indicated that polymyxin B_1_ could enhance the resistance of rice to *Xoo* pathogen through upregulating CAD and POD in rice.

### Defensive Enzyme Activities

**Figure 9:**
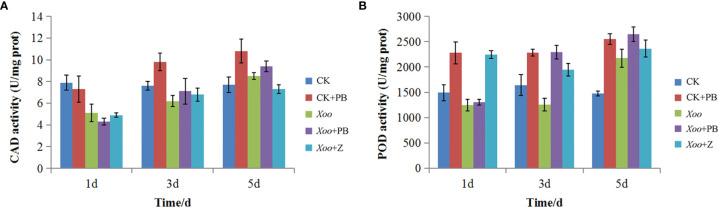
Effects of polymyxin B_1_ (PB) on CAD **(A)** and POD **(B)**k activities in rice leaves. Data presented are averages of pooled data (n = 3). Bars represent standard deviations of the means.

As shown in **Figure 9A**, there was a significant difference of CAD activity between CK + PB and CK groups on the 3rd and 5th days. Meanwhile, CK + PB groups improved 25.8% of CAD activity than that of CK groups on the 5th day. However, there was no significant difference between *Xoo* + PB and *Xoo* groups on the 1st, 3rd, and 5th days. As shown in **Figure 9B**, the enzyme activity of POD significantly increased after treatment with polymyxin B_1_, yielding the maximum on the 5th day. In detail, CK + PB treatment improved 46.5% of POD activity that of CK treatment. In contrast, the significant difference in POD activity between *Xoo* + PB and *Xoo* groups was revealed on the 3rd day at 42.5%. The results showed that polymyxin B_1_ could increase the defensive enzyme activity and then activate the systemic acquired resistance of rice, and inhibit *Xoo* infection.

### PRM Analysis

**Figure 10:**
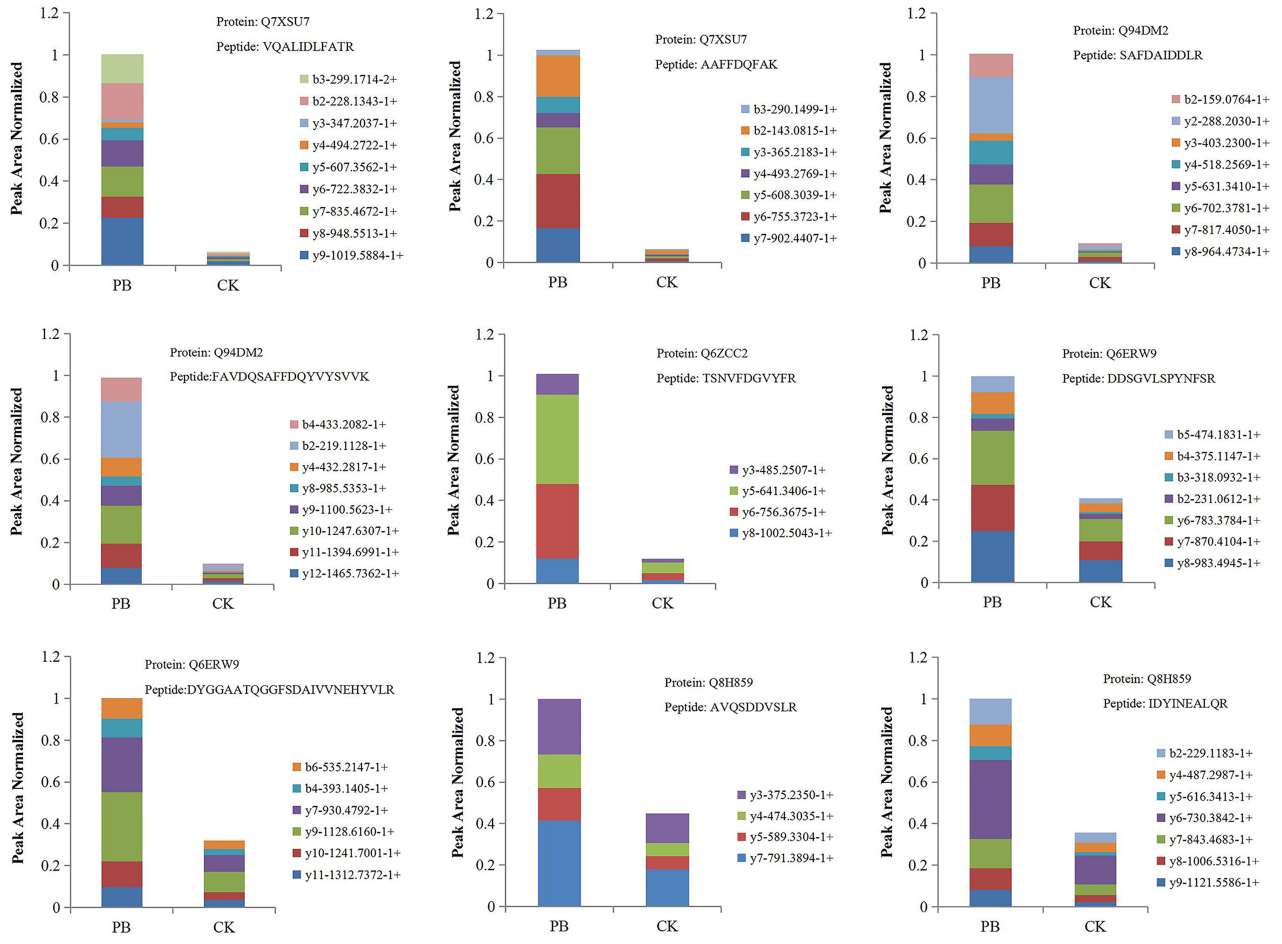
The fragment ion peak area distribution map of unique peptides of some POD and CAD. PB is the *Xoo* + polymyxin B_1_ group and CK is the *Xoo* group of rice.

PRM was performed to confirm the potential relationship of 16 DEPs to the phenylpropanoid biosynthesis pathway. As shown in **Table 4** and **Figure 10**, the expression levels of 16 DEPs were consistent with proteomic analysis results. Three POD proteins were significantly upregulated, namely, Q94DM2, Q6ZCC2, and Q7XSU7. Notably, the expression level of Q7XSU7 increased 17.64-fold after *Xoo* + PB treatment than that of *Xoo* group (PB/CK). Meanwhile, the PB/CK ratio of Q94DM2 and Q6ZCC2 protein was 9.89 and 8.34, respectively. Expression levels of Q8H859 and Q6ERW9 of CAD upregulated 2.34-fold and 2.19-fold versus CK groups. Among DEPs of phenylpropanoid biosynthesis pathway, A0A0P0V2C2, Q9AS12, and A2Y375 were downregulated. The PRM results demonstrated that polymyxin B_1_ could significantly affect the expression level of POD and then regulate the phenylpropanoid biosynthesis pathway, which were consistent with DEPs analysis.

**Table 4:** PRM protein quantification results.

Protein Accession	Gene name	CK Relative Abundance	PB Relative Abundance	PB/CK Ratio	PB/CK *P*-value
A0A0N7KI36	Os03g0762300	0.64	1.36	2.11	0.00003
A0A0P0XR31	Os10g0109300	0.65	1.35	2.07	0.00005
Q5JMS4	Os01g0962700	0.67	1.33	1.96	0.00223
Q94DM2	prx22	0.18	1.82	9.89	0.00008
A2YPX2	OsI_27326	0.45	1.55	3.42	0.00224
Q6ZCC2	Os08g0113000	0.21	1.79	8.34	0.00002
Q6K4J4	prx122	0.53	1.47	2.74	0.00004
Q7XSU7	Os04g0688500	0.11	1.89	17.64	0.00000
Q7XSU8	Os04g0688300	0.46	1.54	3.31	0.00000
A0A0P0V2C2	Os01g0326300	1.08	0.92	0.85	0.05490
Q9AS12	prx16	1.40	0.60	0.43	0.00009
Q8H859	CAD1	0.63	1.37	2.17	0.00002
Q6ERW9	CAD8B	0.60	1.40	2.34	0.00026
Q6ERW7	CAD8C	0.69	1.31	1.88	0.00043
Q60DX8	BGLU22	0.86	1.14	1.33	0.00900
A2Y375	CYP73A	1.24	0.76	0.61	0.00098

### Conclusions

In conclusion, polymyxin B_1_ and E_2_ were firstly isolated from *P polymyxa* Y-1 and later were evaluated on their antibacterial activities *in vitro* and *in vivo*. Notably, polymyxin B_1_ and E_2_ possessed outstanding inhibitory activities against *Xoo* and *Xoc* through destroying the cell integrity of *Xoo*, reducing its infectivity and enhancing rice resistance to defense pathogen infections. This study implied that polymyxin B_1_ and E_2_ can serve as new microbial pesticides for controlling rice bacterial disease and provide an eco-friendly method for developing new pesticides.

## Data Availability Statement

The original contributions presented in the study are included in the article/**Supplementary Material**. Further inquiries can be directed to the corresponding author.

## Author Contributions

XG and WY conceived and designed the research. XG and WY wrote the manuscript. CC analyzed the data. All authors listed have made a substantial, direct, and intellectual contribution to the work and approved it for publication.

## Funding

This work was supported by the National Key Research and Development Program of China (2018YFD0200100), the Construction Project of Key Laboratories from the Education Department of Guizhou Province (QJHKY[2018]001), the Subsidy Project for Outstanding Key Laboratory of Guizhou Province in China (20154004), and the Program of Introducing Talents of Discipline to Universities of China (111 Program, D20023).

## Conflict of Interest

The authors declare that the research was conducted in the absence of any commercial or financial relationships that could be construed as a potential conflict of interest.

## Publisher’s Note

All claims expressed in this article are solely those of the authors and do not necessarily represent those of their affiliated organizations, or those of the publisher, the editors and the reviewers. Any product that may be evaluated in this article, or claim that may be made by its manufacturer, is not guaranteed or endorsed by the publisher.

## Supplementary Material

Supplementary data includes ^13^C NMR, ^1^H NMR and HRMS of polymyxin B_1_ and E_2_. Antibacterial activity assay of *P. polymyxa* supernatant. LC-MS/MS identified 4431 proteins, which were shown in Appendix LC-MS data.
