# Supplementary material for: Polymyxin B1 and E2 From Paenibacillus polymyxa Y-1 for Controlling Rice Bacterial Disease
Source: Front Cell Infect Microbiol. 2022 Mar 28;12:866357. doi: 10.3389/fcimb.2022.866357 (PMC8995708; doi:10.3389/fcimb.2022.866357)
Supplement: Supplementary file 2 [file Table_2.docx]

Supplementary Material

**1. Antibacterial activity assay of *P. polymyxa* supernatant against *Xoo* and *Xoc*.**

**
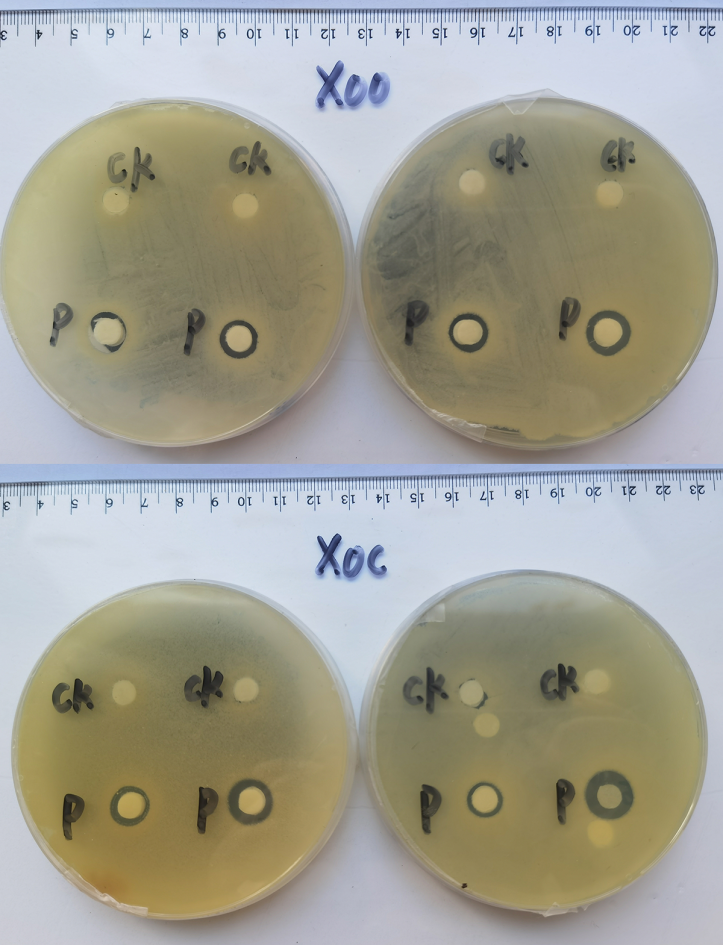
**

**Figure S1** The paper disk method was used to determine the in vitro antibacterial activity of *P. polymyxa* supernatant against *Xoo* and *Xoc*.

**2. Characterization of metabolites Y1 and Y2.**

**Polymyxin B_1_ (Y1)**. Light yellow powder; m.p. 219–221°C. ^1^H NMR (600 MHz, D_2_O) *δ* 7.36 (d, *J* = 5.7 Hz, 2H), 7.32 (d, *J* = 6.2 Hz, 1H), 7.24 (d, *J* = 5.5 Hz, 2H), 4.51 (s, 4H), 4.34 (s, 1H), 4.24 (s, 5H), 4.14 (s, 2H), 3.37 (s, 1H), 3.31–2.98 (m, 11H), 2.97 (d, *J* = 10.6 Hz, 1H), 2.90 (s, 1H), 2.30 (s, 2H), 2.28–1.76 (m, 14H), 1.56 (s, 2H), 1.51–1.36 (m, 2H), 1.33–1.10 (m, 12H), 0.81 (d, *J* = 4.8 Hz, 6H), 0.68 (d, *J* = 5.0 Hz, 2H), 0.60 (s, 2H). ^13^C NMR (151 MHz, D_2_O) *δ* 177.7, 175.1, 173.6, 173.4, 173.1, 173.0, 172.9, 172.4, 171.9, 171.8, 171.5, 135.3, 129.0, 129.0, 128.9, 128.9, 127.4, 67.0, 66.1, 59.7, 59.0, 57.4, 56.3, 53.0, 51.9, 51.6, 51.4, 51.0, 50.4, 38.9, 37.8, 36.4, 36.3, 36.0, 35.4, 35.3, 33.5, 30.3, 29.6, 28.8, 28.5, 28.2, 27.9, 27.1, 26.0, 25.7, 25.5, 23.1, 22.4, 21.9, 20.2, 19.1, 18.8, 18.5, 16.8, 10.7. HRMS (ESI): calculated for C_56_H_99_N_16_O_13_ [M+H]^+^: 1203.75720, found: 1203.69739.

**Polymyxin E_2_ (Y2)**. Yellow powder; m.p. 216–218°C. ^1^H NMR (600 MHz, D_2_O) *δ* 4.55–4.50 (m, 3H), 4.41–4.35 (m, 3H), 4.30–4.22 (m, 5H), 4.18 (d, *J*= 4.78 Hz, 1H), 3.40–3.34 (m, 1H), 3.22–2.99 (m, 11H), 2.33–2.31 (m, 2H), 2.27–1.97 (m, 11H), 1.92–1.87 (m, 1H) 1.70–1.49 (m, 9H), 1.30–1.27 (m, 2H), 1.22–1.12 (m, 8H), 0.94–0.81 (m, 18H). ^13^C NMR (151 MHz, D_2_O) *δ* 177.7, 175.0, 174.9, 173.5, 173.1, 172.9, 172.9, 172.4, 172.2, 171.9, 171.5, 67.0, 66.2, 59.7, 59.0, 53.3, 53.0, 51.8, 51.5, 51.1, 51.1, 50.6, 39.5, 38.9, 37.8, 36.5, 36.3, 36.1, 35.4, 35.3, 33.5, 30.4, 29.6, 28.8, 28.6, 28.5, 28.1, 27.1, 26.0, 25.7, 25.5, 24.3, 22.5, 21.9, 21.7, 21.1, 19.9, 19.1, 18.8, 18.5, 10.7. HRMS (ESI): calculated for C_52_H_99_O_13_N_16_ [M+H]^+^: 1155.75720, found: 1155.76685.

**3. ^1^H NMR, ^13^C NMR and HRMS of metabolites Y1-Y2.**


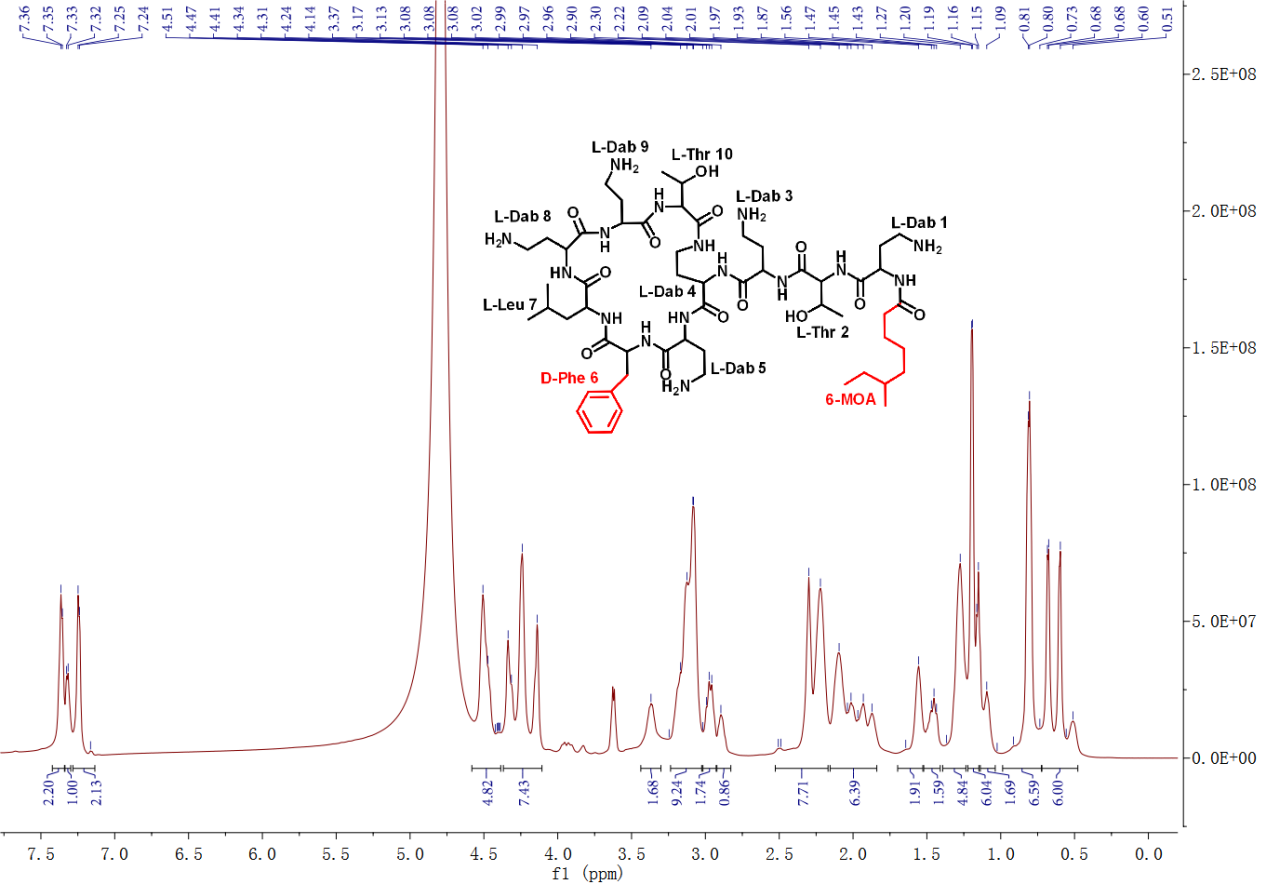


Figure S2 ^1^H NMR of polymyxin B_1_ (Y1)


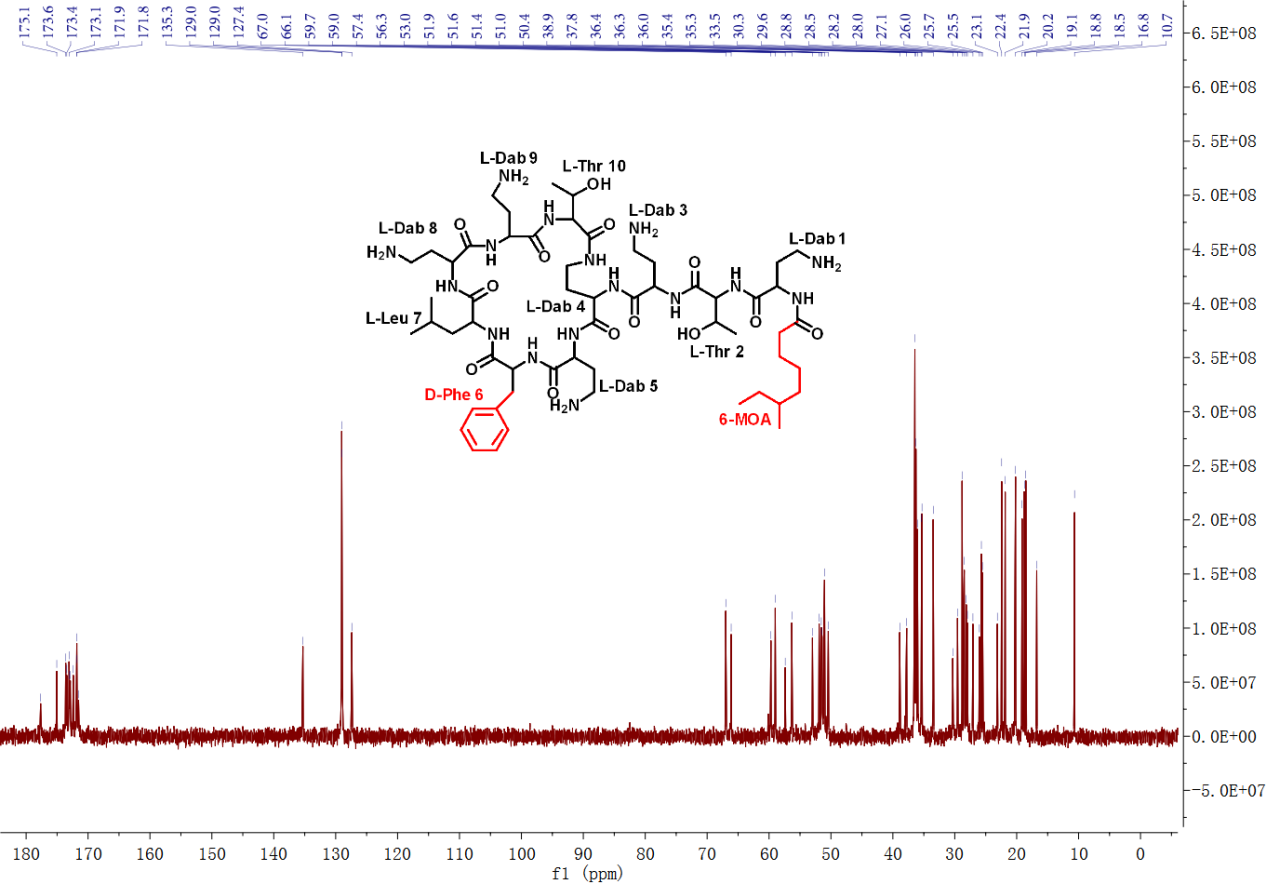


Figure S3 ^13^C NMR of polymyxin B_1_ (Y1)


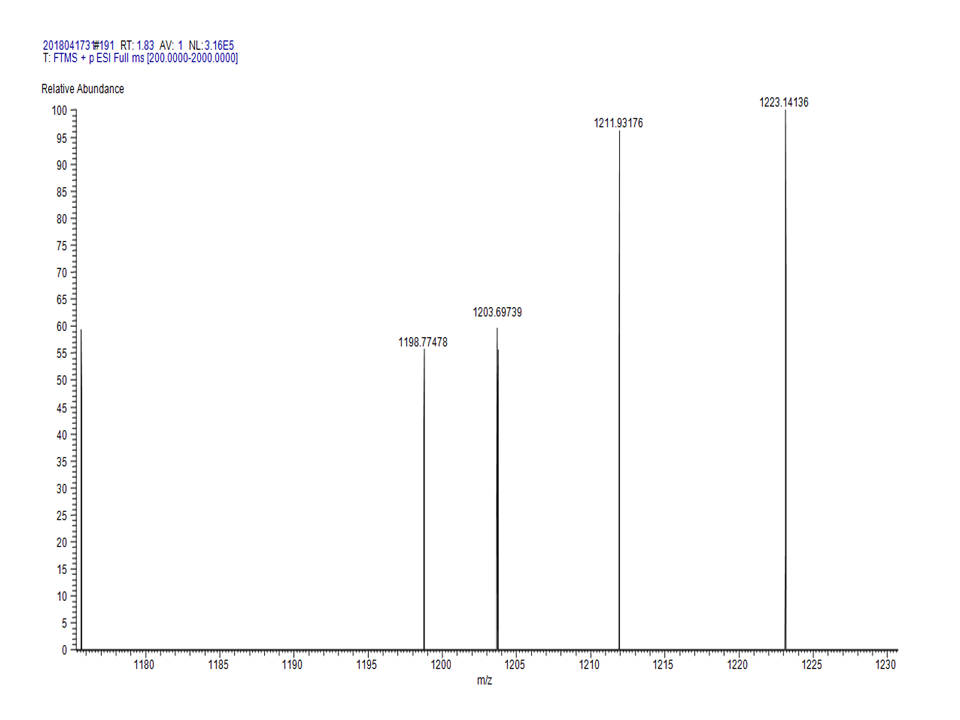


Figure S4 HRMS of polymyxin B_1_.(Y1)


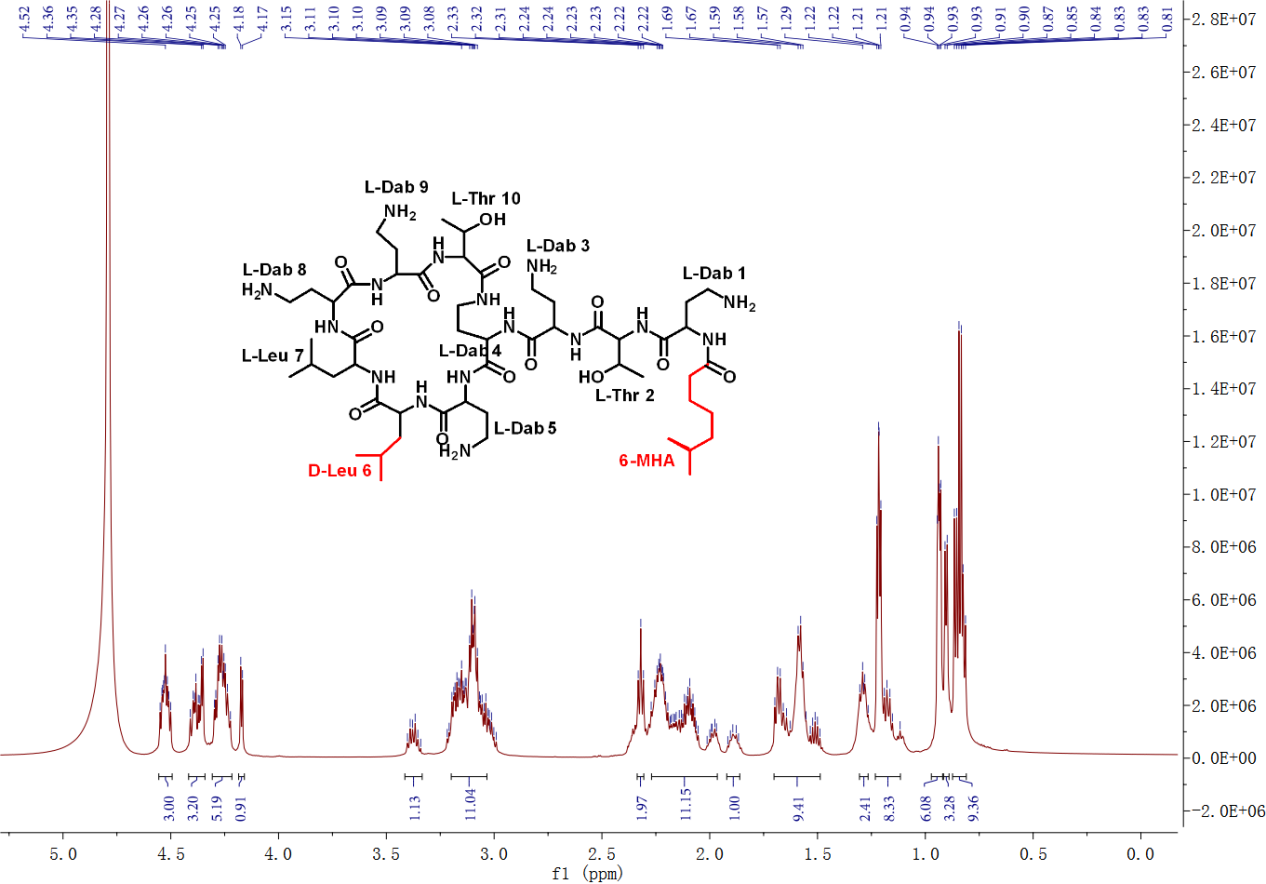


Figure S5 ^1^H NMR of polymyxin E_2_ (Y2)


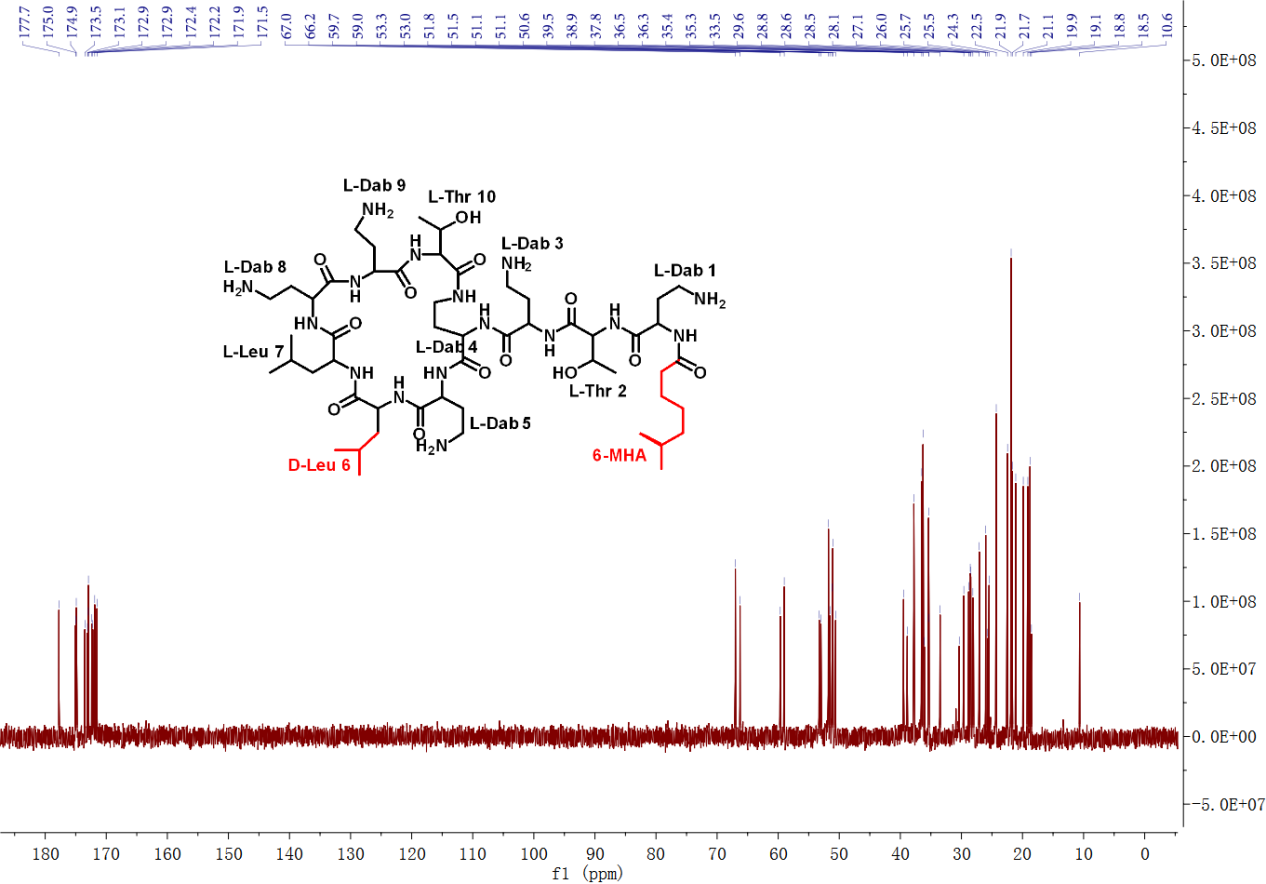


Figure S6 ^13^C NMR of polymyxin E_2_ (Y2)


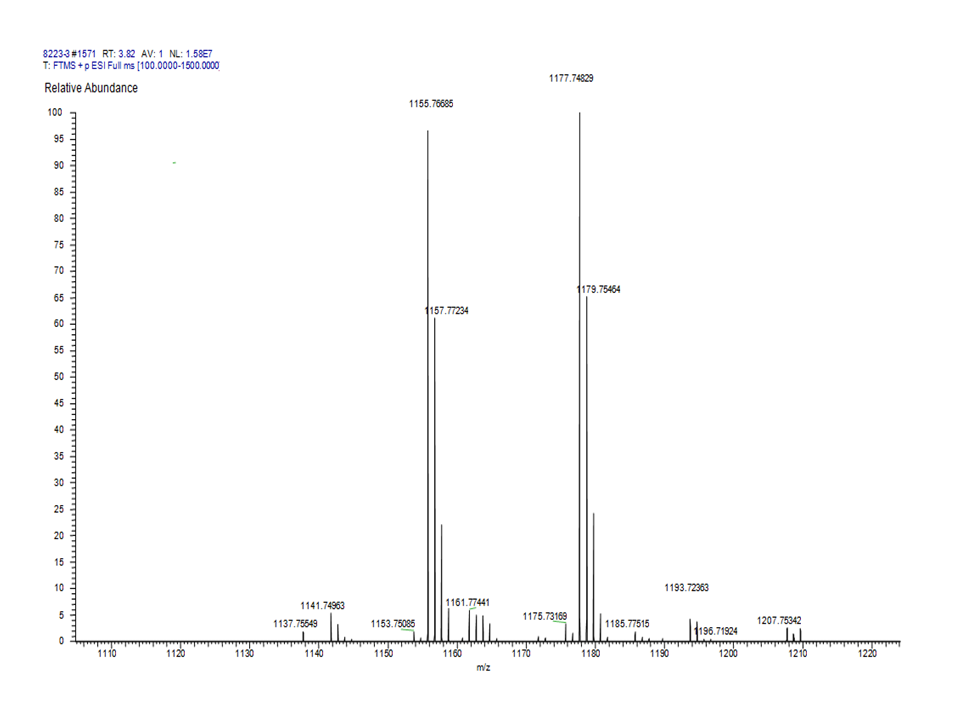


Figure S7 HRMS of polymyxin E_2_. (Y2)
